# Phylogeny, host use, and diversification in the moth family Momphidae (Lepidoptera: Gelechioidea)

**DOI:** 10.1371/journal.pone.0207833

**Published:** 2019-06-06

**Authors:** Daniel J. Bruzzese, David L. Wagner, Terry Harrison, Tania Jogesh, Rick P. Overson, Norman J. Wickett, Robert A. Raguso, Krissa A. Skogen

**Affiliations:** 1 Department of Plant Biology and Conservation, Northwestern University, Evanston, IL, United States of America; 2 Division of Plant Science and Conservation, Chicago Botanic Garden, Glencoe, IL, United States of America; 3 Department of Ecology and Evolutionary Biology, University of Connecticut, Storrs, CT, United States of America; 4 Independent Researcher, Charleston, IL, United States of America; 5 Global Institute of Sustainability, Arizona State University, Tempe, AZ, United States of America; 6 Department of Neurobiology and Behavior, Cornell University, Ithaca, NY, United States of America; National Taiwan Normal University, TAIWAN

## Abstract

Insect herbivores and their hostplants constitute much of Earth’s described biological diversity, but how these often-specialized associations diversify is not fully understood. We combined detailed hostplant data and comparative phylogenetic analyses of the lepidopteran family Momphidae to explore how shifts in the use of hostplant resources, not just hostplant taxon, contribute to the diversification of a phytophagous insect lineage. We inferred two phylogenetic hypotheses emphasizing relationships among species in the nominate genus, *Mompha* Hübner. A six-gene phylogeny was constructed with reared exemplars and collections from hostplants in the family Onagraceae from western and southwestern USA, and a cytochrome c oxidase subunit 1 (COI) phylogeny was inferred from collections and publicly available accessions in the Barcode of Life Data System. Species delimitation analyses combined with morphological data revealed ca. 56 undescribed species-level taxa, many of which are hostplant specialists on Onagraceae in the southwestern USA. Our phylogenetic reconstructions divided Momphidae into six major clades: 1) an Onagraceae flower- and fruit-boring clade, 2) a Melastomataceae-galling clade, 3) a leafmining clade A, 4) a leafmining clade B, 5) a *Zapyrastra* Meyrick clade, and 6) a monobasic lineage represented by *Mompha eloisella* (Clemens). Ancestral trait reconstructions using the COI phylogeny identified leafmining on Onagraceae as the ancestral state for Momphidae. Our study finds that shifts along three hostplant resource axes (plant taxon, plant tissue type, and larval feeding mode) have contributed to the evolutionary success and diversification of momphids.

## Introduction

Phytophagous insects constitute nearly a quarter of described metazoan diversity [1,2], and phytophagy represents the predominant feeding habit in many of the largest insect orders (especially Coleoptera, Hemiptera, and Lepidoptera). The mechanisms underlying the generation and maintenance of this extraordinary diversity remain an active area of inquiry [3,4], and a better understanding of the macroevolutionary processes underlying diversification is essential for protecting much of earth’s biodiversity.

Dietary specialization is a major factor in the diversification of phytophagous insect groups [5–9]. Shifting to a new hostplant niche has the potential to supply underexploited resources, enemy-free space, as well as release from competition [10–12]. The colonization of a new hostplant niche sometimes triggers the process of specialization, whereby processes such as local adaptation, assortative mating, and divergent selection lead to isolation and ultimately speciation [13–17]. In addition to specialization via switches to a novel hostplant taxon, insect specialization and speciation may follow population-level changes in a hostplant’s morphology, chemistry, or phenology [18,19]. Geographic factors also play a strong role in insect speciation [20–23], as populations become isolated by geographic barriers [24] or as a consequence of limited dispersal [25].

To understand macroevolutionary processes operating within and across phytophagous insect lineages, niche shifts have been superimposed onto their phylogenies [22,26–30]. Such assessments support two paradigms of phytophagous insect diversification. The musical chairs hypothesis postulates that diversification occurs when a dietarily specialized insect lineage shifts to a new hostplant taxon, with its host breadth remaining the same [31]. In contrast, the oscillation hypothesis predicts that diversification often arises from the narrowing of an insect lineage’s host breadth, with generalized lineages commonly giving rise to more specialized species [9,32]. While providing a useful framework to understand dietary specialization, these hypotheses focus on a singular aspect of the hostplant resource, its taxonomic component (i.e., the hostplant family, genus, or species). However, specialization can also occur through adaptation to a novel tissue type (e.g., leaves, fruits, or flowers) or larval feeding mode (e.g., chewing, mining, galling, or boring) within the same hostplant taxon [33–35]. The bogus yucca moths (*Prodoxus* Riley) and their *Yucca* L. (Asparagaceae) hosts provide a good example of specialization via both taxonomic and hostplant tissue shifts. Unlike their seed-feeding kindred genus (*Tegeticula* Zeller), *Prodoxus* feed on the sterile tissues of the flower stalks and pods (and rarely leaves) of their *Yucca* hosts. Their diversification is thought to have resulted from colonization of new plant structures as well as shifts to new hostplant species [36,37]. Transitions to novel niche axes of a hostplant resource can be just as important in diversification as taxonomic shifts [16,38–40]. Though, the relative lability, frequency, and dynamic nature of within-host shifts have not received appreciable quantification, especially in a fine-scale phylogenetic context, and doing so would help unravel the processes of phytophagous insect specialization and speciation.

**Fig 1:**
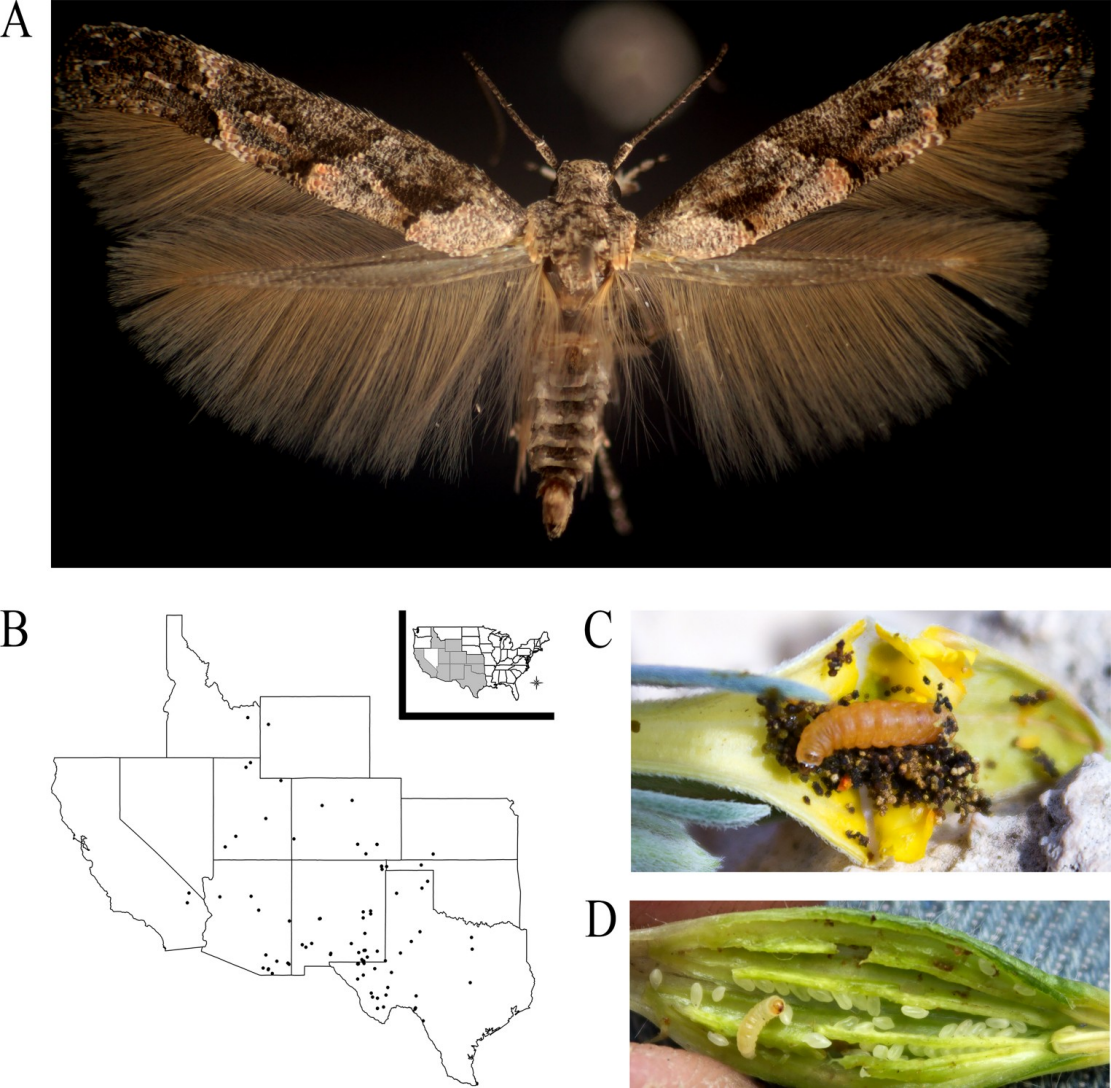
*Mompha* overview. A) *Mompha pecosella* Busck complex, Monahans Sandhills, TX from *Oenothera capillifolia* subspecies *berlandieri* W. L. Wagner & Hoch. Photo: TH. B) Distribution of 87 *Mompha* collections from ten states. C) An opened *Oenothera lavandulifolia* Torr. & A. Gray flower bud showing a *Mompha* larva. Photo: RPO. D) An opened *Oenothera caespitosa* Nutt. fruit showing a *Mompha* larva. Photo: RPO.

The microlepidopteran family, Momphidae (Lepidoptera: Gelechioidea) and its hostplants provide an ideal system to evaluate the role that hostplant resource shifts can have on phytophagous insect diversification. Momphidae are a cosmopolitan family comprising of approximately 120 named species [41] characterized by narrow forewings with raised scale tufts (Fig 1A). Their larvae exploit a sweeping array of hostplant resources by mining, galling, and boring in six plant tissues: flowers, fruits, leaves, shoot tips, stems, and roots. So far as is known, all momphids are monophagous or oligophagous on one of seven dicot families: Cistaceae, Haloragaceae, Lythraceae, Melastomataceae, Onagraceae, Rubiaceae, and Polygonaceae [42–46]. The nominate genus, *Mompha* Hübner, is the largest genus within the family. Much of its described diversity is restricted to Onagraceae [46–49], suspected to have arisen from repeated within-family radiations [35,37]. *Mompha* also exhibit lability in hostplant-tissue utilization between different broods [46] or instars [48]. Recent studies of *Mompha* genitalia and cytochrome c oxidase subunit 1 (COI) sequences indicate that the family is rich in cryptic species and undescribed taxa, and underscore the need for more intensive taxonomic study and a robust assessment of momphid feeding niches, host plant usage, and systematic relationships [47–51].

In this study, we combine phylogenetic reconstruction with detailed hostplant-resource data (host taxonomy, plant tissue type, and larval feeding mode) to examine how shifts across hostplant resource axes have contributed to the specialization and diversification of Momphidae, a poorly studied, but species-rich insect group. We address the following questions: (1) What are the phylogenetic relationships among momphids? (2) What is the ancestral hostplant taxon, tissue resource, and feeding mode of momphids? (3) Are shifts to new hostplant tissues and changes in larval feeding modes as prevalent as shifts to new hostplant family? We examined these questions with two data sets by (1) reconstructing a six-gene phylogeny primarily consisting of a subset of momphids from Onagraceae-feeding *Mompha* from the southwestern USA [52] and other Nearctic momphid species [47,53], and (2) inferring a preliminary global tree of the family using COI barcodes.

## Materials and methods

### Sample collection

A total of 842 momphid samples were included in the analyses. Of these, 131 were collected from 87 hostplant populations in the western and southwestern USA (Fig 1B) (S1 Table, MP accessions) in 2014, 2015, and 2016. The following landowners provided permission and access to field sites: U.S. National Park Service: Carlsbad Caverns NP (CAVE-2014-SCI-0017), Guadalupe Mountains NP (GUMO-2015-SCI-009), and Mojave NP (MOJA-2015-SCI-0013); U.S.D.A. Forest Service: Ashley National Forest (0414), Bridger-Teton National Forest (Jogesh2015), Cache National Forest (0414), Comanche National Grasslands (Jogesh2015), Coronado National Forest (RO-231, #R-e RO 252), Gila National Forest (RO-231, #R-e RO 252), and Madera Canyon (#R-e RO 252); and U.S. Bureau of Land Management: Colorado (6850 (CO-932)), Idaho (1110 (931 AH)), New Mexico (6850 (93000)), and Utah (6840 (UT-933)). Collections focused primarily on hostplants in *Oenothera* L., a large genus in Onagraceae with exceptional diversity in the southwestern USA [52]. Larvae and pupae of momphids were collected from flowers, fruits, leaves, shoot tips, stems, and roots when hostplants were at or near reproductive maturity (Fig 1C and 1D). Collected (larval) momphids were fixed in 100% ethanol and stored at -20°C. Often, a second set of immatures was collected and reared to the adult stage to aid identification efforts.

We randomly selected one individual from each feeding resource axis: hostplant species; hostplant tissue type (flower, fruit, leaf, shoot tip, stem, and root); and feeding mode (galling, boring, and mining) within a hostplant population to be sequenced. To make sure our sampling scheme did not overlook instances of multiple momphid species occupying the same niche, we barcoded individuals from ten hostplant populations for each hostplant species and resource axis. For each hostplant resource axis within each hostplant population, we found near-identical COI sequences, which suggests that momphid species typically occupy a unique hostplant niche, validating our sampling (sequencing) scheme. An additional 47 adult momphids were acquired for sequencing from the T. Harrison collection (Charleston, IL USA, S1 Table, TH accessions)—many of these accessions had been identified to species by TH based on morphological characters, including those of the genitalia. For further assessment of taxonomic relationships, published COI barcodes for 664 momphid accessions, representing three continents and 15 countries (S1 Table), were downloaded from the Barcode of Life Data System (BOLD) (http://www.boldsystems.org) [54].

### DNA extraction, PCR, and sequencing

For the 178 previously unsequenced samples, DNA was extracted from either the anterior third of a caterpillar or a single adult leg, with a modified Chelex 100 and Proteinase-K protocol [55]. We amplified partial coding sequences from one mitochondrial locus (COI) and five nuclear loci [glyceraldehyde-3-phosphate dehydrogenase (GADPH), elongation factor 1-alpha (EF-1α), dopa decarboxylase (DDC), carbamoyl phosphate synthase domain protein (CAD), and histone 3 (H3)] (S2 Table). These loci have been used to reconstruct species-level relationships in other phytophagous insect genera [56–63]. PCR was performed in 10 μL volumes. Thermocycler programs were optimized for each primer pair. PCR product was verified with a 1% agarose gel stained with SYBR Safe DNA gel stain (Life Technologies, Grand Island, NY, USA) and stored at 4°C. For failed reactions, PCR was reattempted with internal primers generated with Primer-Blast for DDC, CAD, and GADPH (S2 Table) [64]. Successful amplicons were purified using Exonuclease I and Shrimp Alkaline Phosphatase (Affymetrix). Purified PCR product was sequenced in the forward direction using a modified 10 μL BigDye Terminator v3.1 (ThermoFisher) cycle sequencing reaction with standard thermocycler conditions. Sequenced product was purified with an EtOH/EDTA cleanup and read on an ABI 3730 DNA Analyzer. Additional details and protocols are given in the S1 Appendix.

Two datasets were generated for phylogenetic reconstruction: (1) momphids collected in the USA, Costa Rica, and New Zealand and sequenced for six genes (MP and TH *Mompha*), totaling 178 samples (six-gene dataset); (2) Pooled BOLD COI accessions and COI sequences from the six-gene dataset, totaling 842 individuals (COI dataset). Six outgroup species were selected from two recent phylogenetic analyses [61,63]. Five of the six outgroup taxa fall within the scythridid assemblage: *Batrachedra praeangusta* (Haworth), *Blastodacna hellerella* (Duponchel), *Coleophora caelebipennella* Zeller, *Hieromantis kurokoi* Yasuda, and *Hypatopa binotella* Thunberg, and one is from Gelechiidae: *Exoteleia dodecella* (L.).

### Phylogenetic analyses

For each locus, sequence chromatograms were converted to FASTA format in UGENE [65] and aligned at the nucleotide level with the FFT-NS-I algorithm in MAFFT v. 7.308 [66]. Aligned chromatograms were error-checked in parallel with the design tool in Genome Compiler (http://www.genomecompiler.com/). Low-quality base calls were corrected with IUPAC nucleotide ambiguity codes in AliView [67]. AliView also verified open reading frames in amino acid alignments and trimmed sequences to equal length: CAD: 615 bp, COI: 558 bp, DDC: 288 bp, EF-1α: 474 bp, GADPH: 582 bp, H3: 276 bp. FASTA files were concatenated with SequenceMatrix v1.8 [68] to generate the six-gene dataset. Gene sequences were uploaded to GenBank (S1 Table).

Phylogenies were reconstructed with Maximum Likelihood (ML), Bayesian inference, and multispecies coalescent methods using the CIPRES research computing resource [69]. Best-fitting substitution models and partitions for MrBayes and RAxML were selected with Bayesian Information Criterion (BIC) in PartitionFinder v1.1.1 [70]. We fitted partitions and substitution models for both whole genes and codon positions to avoid overfitting (S3 Table). For the six-gene and COI datasets, ML analyses were performed with RAxMLv8.2.9 [71] with 10 ML starting tree searches and 1000 bootstrap replicates using the inferred partitioning schemes and substitution models from PartitionFinder.

For both the six-gene and COI datasets, Bayesian analyses were carried out using MrBayes v3.2.6 [72]. For inferred partitioning schemes and substitution models, two independent runs were executed with three heated chains and one cold chain for 10 million generations sampled every 1000 generations. For the six-gene dataset, coalescent analysis was performed with four independent runs of StarBEAST2 [73], with bModelTest [74], unlinked strict estimated clocks for each locus, and default Yule priors for 150 million generations sampled every 5000 steps. For the COI dataset, BEAST2 v2.3.2 [75] was used to estimate relative divergence times of momphid lineages. Four independent runs were executed with bModelTest, a strict molecular clock, and default Yule priors for 40 million generations sampled every 1000 steps. Convergence for each Bayesian and coalescent analysis was evaluated in TRACER [76] checking for ESS values greater than 200. If trees from an analysis converged, resulting trees were combined with a 10% burn-in and thinned to 10,000 trees with the program LogCombiner. Finally, a maximum clade credibility tree was generated for each analysis using median ancestor heights with the program TreeAnnotator. Agreement between tree topologies from ML, Bayesian, and coalescent analyses for each dataset was evaluated using the APE [77] package in RStudio v3.3.1 [78] and FigTree v1.4.3 [79].

### Species delimitation

Morphological identification of reared individuals from the TH dataset were combined with three commonly used molecular species delimitation analyses to assign taxa to momphid lineages: (1) Discriminant Analysis of Principal Components (DAPC) [80], (2) Generalized Mixed Yule Coalescent (GMYC) [81], and (3) Poisson Tree Processes (PTP) [82]. These analyses were performed once for each dataset using the six-gene MrBayes tree and the COI BEAST tree. DAPC is a multivariate analysis that groups similar individuals and uses aligned sequence data and the R package Adegenet [83]. GMYC is a likelihood method that searches for distinct genetic groups by comparing branching events within and among species using an ultrametric tree and the SPLITS R package [84]. PTP is also a likelihood method that delimits species by using the number of substitutions in branching events to determine interspecies and intraspecies boundaries; it is run with rooted phylogenetic trees. Three implementations of PTP were run: single rate PTP, Bayesian PTP, and Multi-rate PTP [85]. Trees were trimmed to reflect delimited taxa. We assigned unknown momphids names that included information about their natural history. Using “*M*. sp. *Conostegia*.apical.gall.CenAmer.1|TH138” as an example, the designation includes (1) the taxonomy so far as known, here an undetermined species of *Mompha* (*M*. sp.), (2) the hostplant (*Conostegia*), (3) the larval mode of feeding (apical.gall), (4) the geographic origin of the specimen (CenAmer), (5) a postscript to differentiate this moth from any other Central American apical gall inducers on *Conostegia* (1), and (6) a collection number (|TH138).

### Character mapping

To investigate shifts in the utilization of momphid hostplant resources, hostplant tissue type (flower, fruit, leaf, shoot tip, stem, and root), momphid feeding mode (galling, boring, or mining), and hostplant family (Cistaceae, Haloragaceae, Lythraceae, Melastomataceae, Onagraceae, Polygonaceae, and Rubiaceae) were coded onto the tips of the trimmed six-gene and COI phylogenies using GGTree [86] (See S1 Table for character states). Evolution of these discrete traits was traced with stochastic character mapping using 10,000 replicates with the R package Phytools [87]. For stochastic character mapping, tips not associated with hostplant feeding data were coded as “unknown.” While shifts to or from this unknown state may not be actual shifts, coding these shifts can improve the understanding of changes from the previous state.

## Results

### Phylogenetic trees

**Fig 2:**
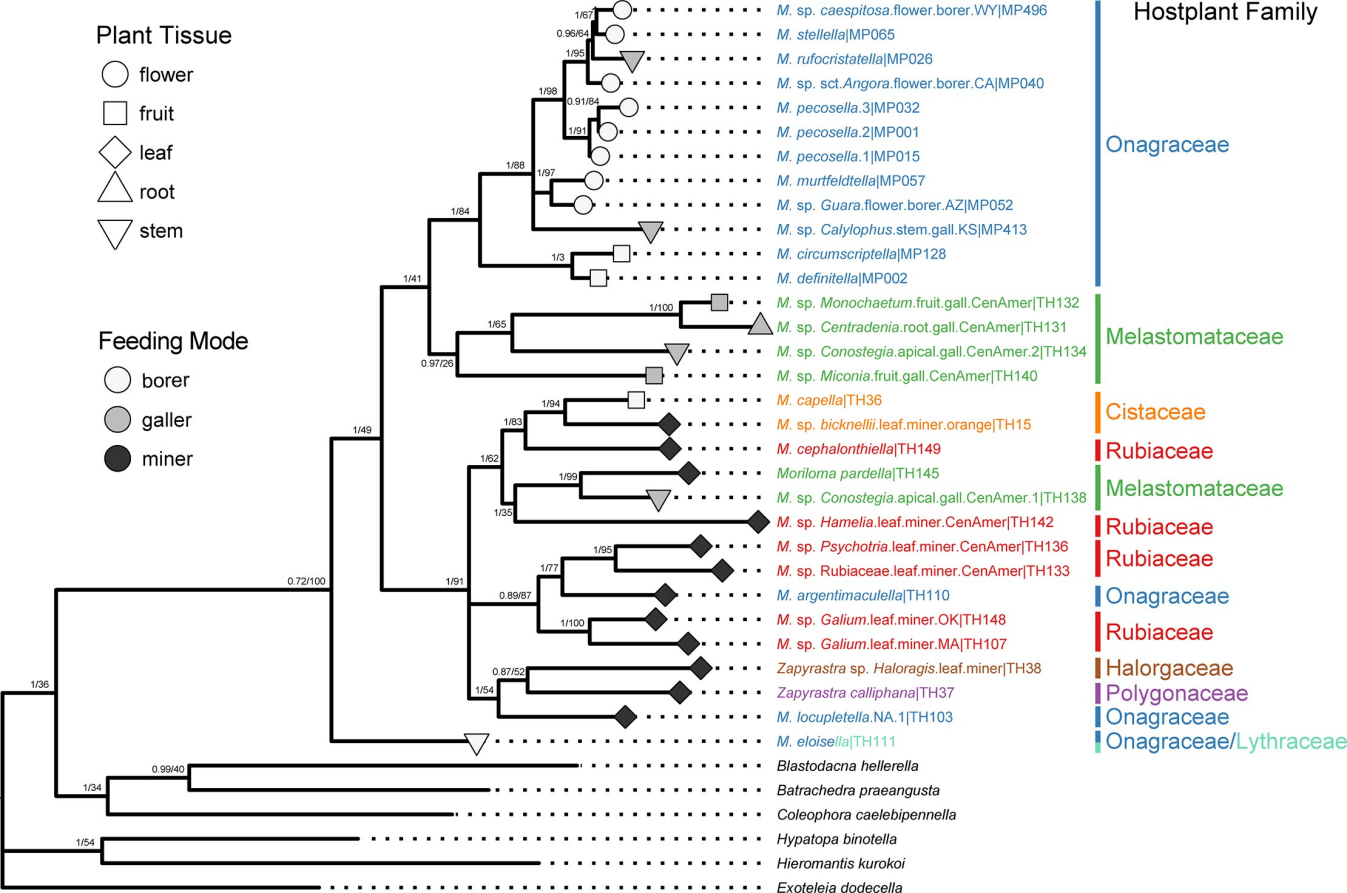
Bayesian phylogeny of the six-gene dataset. The phylogeny is trimmed to reflect the result of species delimitation with one species-level taxon per tip with both posterior support and ML bootstrap support shown at each node (Bayesian posterior: 0–1 / Bootstrap: 0–100). When known, each tip was coded with a symbol for hostplant family, hostplant tissue, and larval feeding mode.

The six-gene dataset consisted of a combined maximum of 2793 bp for 178 ingroup individuals: 150 (84%) of these were sequenced for all six markers and only one (~0.5%) individual was sequenced for fewer than four markers (S1 Table). ML and Bayesian phylogenies from the six-gene dataset, regardless of partitioning scheme, had congruent topologies, with robust support at shallow nodes. The coalescent phylogeny converged, but support across much of the phylogeny was low (<0.75). Nevertheless, the topology from the coalescent phylogeny is largely consistent with the inferred ML and Bayesian phylogenies (all trees can be found in Dryad at:10.5061/dryad.3n1g4td). Molecular delimitation analyses for the six-gene dataset recovered an estimated range of 21–37 momphid species-level taxa (S4 Table): GMYC: 24 taxa; DPAC: 36 taxa; PTP: 30 taxa; bPTP: 37 taxa; and mPTP: 21 taxa (output for each model can be found in Dryad at: 10.5061/dryad.3n1g4td). Morphological-based identification of momphid exemplars combined with molecular delimitation, yielded approximately 31 species-level taxa (Fig 2). Seventeen species-level taxa were recognized as undescribed: of these, ten were collected in Central America and seven from the southwestern USA.

**Fig 3:**
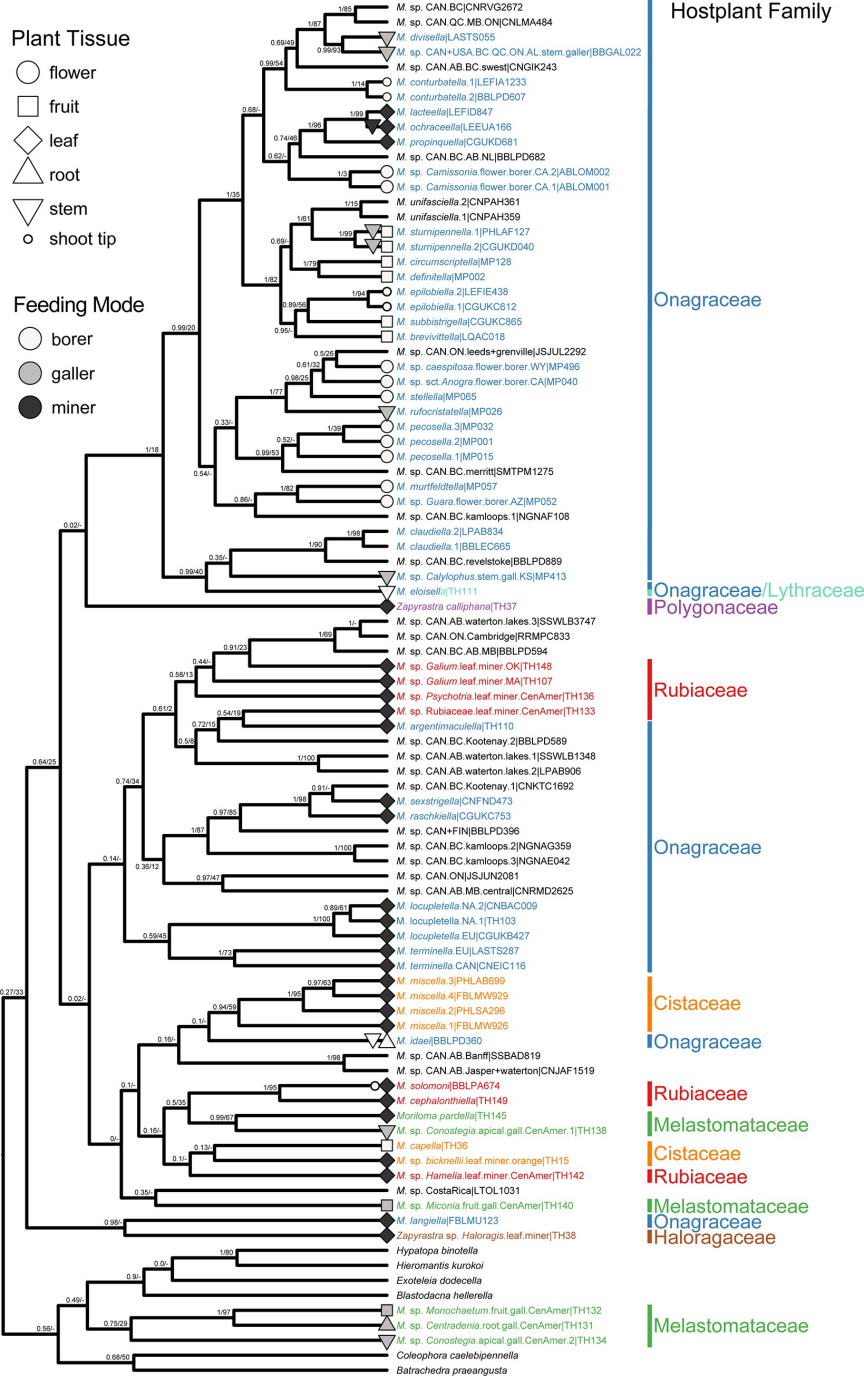
BEAST reconstruction of the COI dataset. The tree is trimmed to reflect the result of species delimitation with one species-level taxon per tip with both posterior support and ML bootstrap support shown at each node (Bayesian posterior: 0–1 / Bootstrap: 0–100). When known, each tip was coded for hostplant family, hostplant tissue, and larval feeding mode.

The combined COI dataset contained a maximum of 558 bp for 842 momphid individuals. ML and Bayesian trees had similar, but non-congruent topologies with often poor support for deeper nodes, and in some cases shallow nodes (trees can be found in Dryad at: 10.5061/dryad.3n1g4td). With its many taxa, the COI tree provides a useful survey of *Mompha* diversity. However, because the COI tree is only reconstructed with a single mitochondrial gene, it contains a few phylogenetic relationships that we believe to be spurious. For example, in the COI phylogeny, three melastome feeders cluster with outgroup taxa, but in the better-supported six-gene phylogeny, they fall within the ingroup. The BEAST reconstruction was selected to represent the COI dataset because it is ultrametric and most similar to the better-supported phylogeny of the six-gene dataset. Because there were incongruences between the two phylogenies, we referred to the greater clade support in the six-gene dataset to resolve conflicts in topology. Molecular delimitation for the COI dataset recovered a range of 79–127 momphid species-level taxa (S5 Table): GMYC: 81 taxa; DPCA: 86 taxa; PTP: 109 taxa; bPTP: 127 taxa; and mPTP: 79 taxa (output for each model can be found in Dryad at: 10.5061/dryad.3n1g4td). Available morphological species identifications from exemplars in the six-gene dataset as well as distributional data were combined with molecular delimitation analyses to conservatively estimate 86 *Mompha* species-level taxa in the COI dataset. Of these, we find corroboratory evidence for 56 undescribed taxa, most of which are endemic to northern latitudes, especially to southwestern USA (Fig 3).

### Ecological characters and phylogenetic relationships within Momphidae

The six-gene phylogeny (Fig 2) supports six major clades of momphids: (1) an Onagraceae flower- and fruit-boring clade, (2) a Melastomataceae-galling clade, (3) a leafmining clade A, (4) a leafmining clade B, (5) a *Zapyrastra* Meyrick clade, and (6) a monobasic lineage represented by *Mompha eloisella* (Clemens). The COI tree (Fig 3) recovers the same general relationships to those of the six-gene phylogeny, but there are incorrect relationships as well, for example the Melastomataceae-galling clade is not recovered (three of its four members cluster with outgroup taxa).

**Fig 4:**
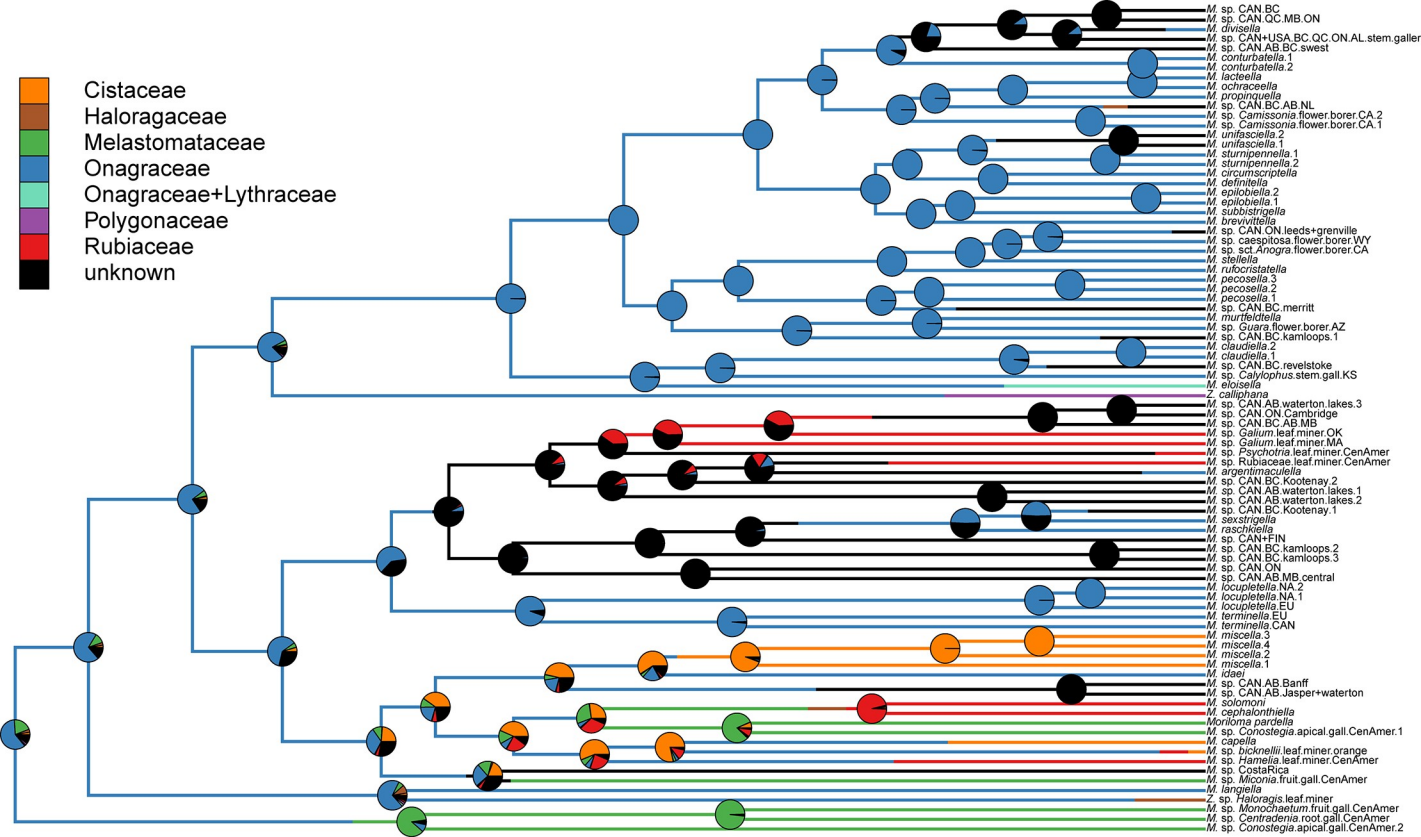
Ancestral trait reconstruction for momphid hostplant family. Stochastic character mapping with 10,000 replicates. Posterior probabilities of ancestral states at each node are displayed as pie charts. Branch colors represent inferred hostplant family history.

Ancestral trait reconstruction identified Onagraceae as the ancestral hostplant family (Fig 4). Shifts from Onagraceae to an undefined host family states were most common (S6 Table). The COI tree suggests that there have been ten shifts to new hostplant families: three to Rubiaceae, two to Cistaceae, two to Melastomataceae, one to Haloragaceae, one to Lythraceae, and one to Polygonaceae. Momphid larvae mine leaves on five hostplant families (Cistaceae, Haloragaceae, Onagraceae, Polygonaceae, and Rubiaceae), induce galls on plant tissue in two hostplant families (Melastomataceae and Onagraceae), and bore into plant tissue of three hostplant families (Cistaceae, Lythraceae, and Onagraceae) (S7 Table). Galling and boring taxa have taxonomically restricted host associations, often feeding on a single hostplant species (S7 Table). Flower-boring arose twice and is restricted to Onagraceae: once in *Camissonia* Link and once in *Oenothera*. Shoot boring arose three times, twice in Onagraceae and once in Rubiaceae. Fruit boring arose twice, once each in Cistaceae and Onagraceae.

**Fig 5:**
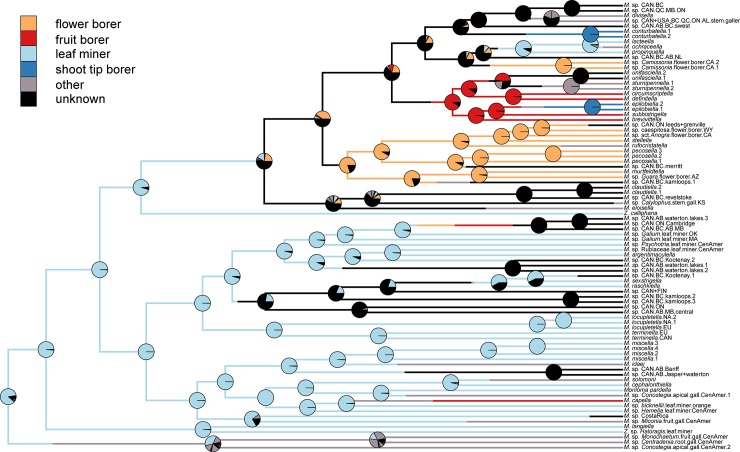
Ancestral trait reconstruction for momphid hostplant resource. Stochastic character mapping with 10,000 replicates. Posterior probabilities of ancestral states at each node are displayed as pie charts. Branches and pie charts are colored coded to the most likely character state at each node. Because of the many character states (15) found in the hostplant resource, we colored the most prevalent states and placed the remaining states in the “other” category.

Ancestral trait reconstruction identified leafmining as the ancestral hostplant resource (Fig 5). Additional analyses that separate feeding mode and plant tissue type confirm that mining was the ancestral feeding mode and that leaves were the ancestral feeding tissue type (S1 and S2 Figs). Eleven independent shifts from mining to new feeding modes occurred on the inferred tree. The most common feeding mode shifts were from boring to unknown, mining to unknown, mining to boring, and from mining to galling (S8 Table). The COI tree suggests that momphids shifted to new hostplant tissue in 17 instances. Shifts from leaf to unknown hostplant tissue, from flower to unknown hostplant tissue, and from leaf to stem + leaf were most prevalent (S9 Table). The Onagraceae-boring clade and the Melastomataceae-galling clade had the highest observed rates of switching among hostplant tissue types (Fig 2).

Momphid taxa range from specialists to oligophagous feeders with some momphids able to consume multiple hostplant species, sections, or sometimes genera, and rarely families (within a single host family) (S7 Table). Some momphids exhibit considerable lability in their use of hostplant resource axes (S7 Table). We identified four instances where a single species exploits two different tissue types: larvae of *Mompha sturnipennella* (Treitschke) galls stems the first generation and bores into fruits in the second generation. In spring, *Mompha solomoni* Wagner, Adamski, and Brown are shoot tip borers and leafminers, but are only leafminers in their summer and fall broods. Early instars of *Mompha ochraceella* (Curtis) mine stems of their hosts, while later instars mine leaves. *Mompha idaei* (Zeller) larvae bore into both the stems and roots of their hosts.

## Discussion

Our phylogenetic reconstructions and species delimitation analyses revealed many new momphid clades and a surprisingly large number of unrecognized species. The six-gene phylogenetic reconstruction of Momphidae (n = 180 exemplars) recovered six well-supported clades: an Onagraceae-flower and fruit-boring clade, a Melastomataceae-galling clade, a leafmining clade A, a leafmining clade B, a *Zapyrastra* clade, and a monobasic lineage represented by *Mompha eloisella*. We anchored our trait-evolution mapping efforts to the better-supported five nuclear- and one mitochondrial-gene phylogeny. Using host association data, we were able to infer that leafmining on an Onagraceae hostplant was the most likely ancestral larval niche for the family. By comparing feeding niche shifts across the 31 momphids in the six-gene phylogeny, we show that shifts across three different hostplant resource axes (host taxon, plant tissue type, and larval feeding mode) all contributed to the specialization and diversification of Momphidae.

We explored COI haplotype diversity across 842 exemplars representing the major lineages within the family. Molecular delimitation programs for identifying species-level taxa yielded estimates of 79–127 momphid species. Based on available morphological, life history, and distributional data, we conservatively estimate that 86 momphid species were represented among our samples, and that 56 of these are currently undescribed species. Most of the new taxa are endemic to northern latitudes, especially to the southwestern USA, of which two-thirds are Onagraceae feeders.

### Host-mediated taxonomic diversification

Our examination of three hostplant resource axes suggested that momphid specialization coincided with shifts to new hostplant taxa, new plant tissue types, and / or changes in feeding modes. While many phytophagous moth lineages undergo diversification via hostplant family shifts [36,88], momphids are notable because their diversification is additionally linked to shifts to new feeding modes and plant structures (that do not also involve a hostplant family change). From their inferred ancestral state of feeding on Onagraceae, momphids more often shifted to a new plant tissue type, rather than to a new host family or feeding mode. Shifts to new hostplant tissue types were restricted to just a few momphid clades. For example, flower boring only developed within the Onagraceae flower- and fruit-boring clade, and appears to have evolved twice, once in *Oenothera*-feeding momphids, and then independently in *Camissonia*-feeding momphids. Shoot boring arose three times, twice in Onagraceae-feeding momphids and once in Rubiaceae-feeding momphids. Fruit boring arose twice, once each in Onagraceae-feeding momphids and in Cistaceae-feeding momphids. The high rate of momphid hostplant shifts coincides with their ability to partition their hostplant resource into microhabitats, as do aphids [21] and *Blepharoneura* Loew flies [89].The six-gene phylogeny shows that shifts to unrelated hostplant families are rare and instead taxonomic shifts have been largely a matter of colonizing new hostplant congeners within Onagraceae.

Our data are relevant to the ongoing debate about the relative importance of the musical chairs versus the oscillation hypothesis in generating herbivore species diversity [9,31,32]. Nearly all momphids are hostplant specialists, feeding on members of a single host genus or species group. The weight of our data suggests that the great diversity of this family has come about through hostplant niche switching within hostplant lineages (e.g., within Onagraceae), with specialists begetting specialists (musical chairs hypothesis), rather than dietary generalists that made host switches across lineages, followed by subsequent trophic specialization of daughter species (oscillation hypothesis). A signal for the existence of dietary generalists across the Momphidae is lacking. Novel host expansions (i.e., shifts to distantly related hostplant families) central to the oscillation hypothesis, and important to generating taxonomic diversity in general, appear to have been modest within Momphidae. Rather, much of the taxonomic diversification within the family appears to be happening “in-house,” i.e., within the Onagraceae, through host, tissue, and feeding-mode switching, further fueled by the diversity of their Onagraceae hosts (see also below). No doubt, other ecological drivers for Momphidae host switching (besides hostplant niche diversity) have played a role in the diversification of the family. For example, interspecific and intraspecific competition and enemy avoidance (from ants and parasitoids) could reinforce shifts to new hostplant tissue taxa and / or larval feeding modes.

Although we highlight the importance of ecological shifts along three hostplant resource axes, other evolutionary forces are also likely to have generated momphid diversity. Both allochrony, and more prominently allopatry, without shifts in hostplant resources, contribute greatly to insect diversity [20,23,90]. The bud-boring *M*. *pecosella* Busck species complex and its hostplant ranges may provide an example. One member of the complex feeds exclusively on *Oenothera toumeyi* (Small) Tidestr., which is widely distributed in Sonora and Chihuahua with a few disjunct populations in the USA, above 1500 m in the Madrean “sky island” mountain ranges of southeastern Arizona. A second *M*. *pecosella* lineage is only known from Utah and Colorado, even though its hostplants, *O*. *lavandulifolia* Torr. & A. Gray and *O*. *pallida* Lindl, are broadly distributed across the southwestern and western USA. The third member of the complex feeds on several *Oenothera* species exclusively in *Oenothera* section *Calylophus* Spach across Texas and New Mexico. None of the three lineages are known to have overlapping ranges, which suggests that allopatry played a role in the diversification of this complex. Allopatry and host resource shifts are likely interwound. For specialization to occur, the new hostplant resource must co-occur and be biologically similar enough to support a hostplant shift, as reported for the bogus yucca moth *Prodoxus decipiens* Riley [34]. Unfortunately, we do not yet have the data necessary to examine how ecological availability of hostplants has led to diversification in *Mompha*, because host breadths are still incompletely known for many members of the Momphidae. Our sampling, while extensive, was both spatially and temporally limited.

### *Mompha* and onagraceae

The COI tree shows that much of the known *Mompha* diversity is associated with Onagraceae, and especially within *Oenothera*, *Epilobium* (L.), and *Chamaenerion* Ség. Our phylogenies document many instances where two or more *Mompha* species share a hostplant. We found three sympatric (and synchronic) *Mompha* species on *Oenothera capillifolia* subspecies *berlandieri* W. L. Wagner & Hoch—each occupying a separate niche: a stem-boring species, *M*. *rufocristatella* (Chambers); a flower-boring species, *M*. *pecosella*; and an undescribed stem-galling species, *M*. sp. *Calylophus*.stem.gall.KA. Host sharing was also observed on *Oenothera biennis* L. where *M*. *stellella* Busck bores in flower buds while *M*. *brevivittella* (Clemens) feeds in developing fruits. The two moths occur sympatrically and are active at the same time of year [91]. These host-sharing instances are suggestive that internal feeding, where the host becomes both the food and the larva’s environment, has led to an increase in hostplant specialization in momphids, as well as other phytophagous insects [42,92,93].

*Mompha* and their Onagraceae hosts have formed close ecological relationships in which *Mompha* are demonstrated to impact hostplant fitness [94] and the evolutionary trajectories of their hostplants at the population level [95,96]. As in other phytophagous insect systems [90,97–99], plant phenology may be a driver of momphid specialization. Because many *Mompha* are strictly dependent on reproductive structures of *Oenothera* for larval survival, adult eclosion must be synchronized with reproductive periods of their hostplants, which may be especially important in arid areas where precipitation and temperature–essential for modulating the timing of flowering [100,101] are commonly unpredictable. Hostplant allelochemicals can also influence phytophagous insect specialization [102,103]. Onagraceae have a diverse and rapidly evolving suite of defensive compounds [104] and this chemical diversity may determine the likelihood of a shift to other taxa or hostplant structure. Regardless of the evolutionary driver, our data suggest that the elevated species richness of *Oenothera* across the southwestern USA, with ca. 75 species occurring within this region [105], set the stage for the diversification of *Mompha*. As shown in other herbivores, herbivore diversity often mirrors hostplant diversity and is widely regarded to promote diversification [9,106,107]. Two-thirds of the 86 species-level taxa in the COI data set (n = 57), and two-thirds of the 56 undescribed species (n = 36) found over the course of this study are believed to feed on Onagraceae. Calibrated phylogenetic analyses of *Oenothera* and *Mompha* will be needed to determine the relative timing and rates of their respective radiations, with the expectation that momphids, like other insect herbivores, lag behind the diversification of their hosts [108]. Based on published phylogenies, *Mompha* is estimated to have split from *Hypatopa* ~80 Ma [109], whereas the estimated age for Onagraceae is ~85 Ma ago [110].

### Taxonomic considerations

The six-gene dataset placed two presumed outgroup taxa within *Mompha*. *Moriloma pardella* Busck (a monotypic genus) nested deeply in a leafmining *Mompha* clade. The type species of *Zapyrastra*, *Z*. *calliphana* Meyrick, also clustered within *Mompha*. Though the family needs taxonomic revision, we refrained from making formal taxonomic recommendations until additional taxa (and genes) can be sampled, especially from the tropics and Southern Hemisphere. While some momphid species are regarded to have Holarctic distributions, our CO1 data suggest that at least some of these instances represent species complexes, e.g. both *M*. *locupletella* (Denis & Schiffermüller) and *M*. *terminella* (Westwood) appear to include distinct North American and European lineages. Focused collaborations between European and North American momphid experts could help resolve the species-level taxonomy in such cases, as was recently achieved for noctuid moths [111].

There remain many undescribed momphid species in the Nearctic, Palearctic, and Neotropical regions. In the Nearctic, where our study was anchored, there are currently 46 recognized momphid species [112]. Our six-gene phylogeny suggests that another seven undescribed species of momphids occur north of Mexico, bringing the total to 53 Nearctic *Mompha* species. The average moth, microlepidopteran, and gelechioid genera in the Nearctic (north of the USA—Mexican border) contain 5.2, 5.8, and 9.3 species, respectively [112]. By any of these measures, *Mompha* represents a speciose genus. Moreover, the estimate of seven undescribed Nearctic species is undoubtedly conservative given that our COI phylogeny identified an additional 33 undescribed Nearctic species-level taxa, and that many areas, plant communities, and hosts have yet to be sampled. *Clarkia* Pursh, an Onagraceae genus with more than 35 species in western North America, is essentially unsurveyed for momphids. In the Neotropics, momphid diversity remains largely unstudied. We have only identified six species-level momphids that consume Melastomataceae, but melastomes are one of the most taxonomically rich and ecologically abundant Neotropical plant families, with over 150 genera [113] and thousands of species. The largest melastome genus, *Miconia* Ruiz & Pav., contains 1050 estimated species [114] and surely hosts numerous undescribed Momphidae. On the basis of male genital morphology, one of the species in the Melastomataceae-galling clade, *M*. sp. *Monochaetum*.fruit.gall.CenAmer|TH132, belongs to the group of momphid species presently assigned to the genus *Palaeomystella* Fletcher. Perhaps all members of the Melastomataceae-galling clade belong within the genus *Palaeomystella* as all known species are gall inducers on Neotropical melastomes [50,115–117]. Further collections will be needed to better explore *Palaeomystella* but our results suggest that the genus could be widespread in much of the Neotropics.

## Conclusion

We generated the first phylogenetic resources for Momphidae and examined diversification patterns across three hostplant resource axes. We find evidence that shifts to novel hostplant taxa, host tissue types, and larval feeding modes have each generated taxonomic diversity. These results underscore the importance of taking into consideration multiple hostplant resource axes, rather than focusing solely on hostplant taxonomy, when assessing herbivore radiations. Our preliminary six-gene and COI trees provide frameworks to guide further collections and biosystematic studies of *Mompha* and momphids more generally. We identified 56 undescribed momphid taxa, but given the geographically limited nature of our sampling, it is likely that many more momphids await discovery. Collections from Melastomataceae in the Neotropics and Onagraceae in Mexico and other areas globally are sure to yield new taxa. Calibrated reconstructions of both *Mompha* and its hostplants would facilitate studies to compare rates of diversification across the genus, map ecological changes, and better elucidate the role that different hostplant niche axes have had in the diversification of Momphidae, and thereby help to unravel and explain the exceptional diversity of this family, and its nominate genus.

## Supporting information

S1 Appendix *Mompha* sequencing protocols. Contains protocols used for DNA extraction, PCR, cleanup, and sequencing of samples.
(DOCX)

S1 Table Momphidae collection metadata. Contains collection information for all *Mompha* specimens represented in the phylogenetic reconstructions. Included are species names, collection location, hostplant taxa, hostplant resource, and GenBank ID. MP accessions denote southwestern collections; TH accessions denote Terry Harrison collections; all other accessions taken from BOLD.
(XLSX)

S2 Table Primer sequence table. Lists loci, primer sequences, and authors.
(XLSX)

S3 Table Inferred partitioning schemes from PartitionFinder. Generated with PartitionFinder using BIC model selection.
(XLSX)

S4 Table Six-gene dataset combined species delimitation results. (XLSX)

S5 Table COI dataset combined species delimitation results. (XLSX)

S6 Table Mean *Mompha* state shifts for hostplant family. Mean is taken from the 10,000 trees used for the ancestral trait reconstruction. Rows are the starting state and columns are the ending state.
(XLSX)

S7 Table Hostplant database for Momphidae. Excel file with known host plant families, host plant species, and larval feeding niches for momphids in this study.
(XLSX)

S8 Table Mean *Mompha* state shifts for feeding mode. Mean is taken from the 10,000 trees used for the ancestral trait reconstruction. Rows are the starting state and columns are the ending state.
(XLSX)

S9 Table Mean *Mompha* state shifts for hostplant tissue. Mean is taken from the 10,000 trees used for the ancestral trait reconstruction. Rows are the starting state and columns are the ending state.
(XLSX)

S1 Fig Ancestral trait reconstruction for *Mompha* larval feeding mode. Stochastic character mapping with 10,000 replicates. Posterior probabilities at each node displayed as pie charts. Colors on branches and the pie charts represent most likely character state at each node.
(TIF)

S2 Fig Ancestral trait reconstruction for hostplant tissue type. Stochastic character mapping with 10,000 replicates. Posterior probabilities at each node displayed as pie charts. Colors on branches and the pie charts represent most likely character state at each node.
(TIF)
