# Supplementary material for: Phylogeny, host use, and diversification in the moth family Momphidae (Lepidoptera: Gelechioidea)
Source: PLoS One. 2019 Jun 6;14(6):e0207833. doi: 10.1371/journal.pone.0207833 (PMC6553701; doi:10.1371/journal.pone.0207833)
Supplement: S1 Appendix — Contains protocols used for DNA extraction, PCR, cleanup, and sequencing of samples. (DOCX) [file pone.0207833.s001.docx]

# S1 Appendix. Sequencing protocols.

**Modified Chelex 100 and Proteinase-K protocol (Casquet et al. 2012)**

1. Place tissue into 1.5 mL microcentrifuge tubes.
2. Add 10ul of Proteinase K (20 mg/ml) to each tube.
3. Add 150ul of 10% Chelex 100 to each tube.
4. Grind specimens with melted pipet tips (acts as a pestle).
5. Incubate for 24 hours at 55 °C., swirling occasionally.
6. Spin down at maximum speed for 10 mins to pellet the Chelex.
7. Pull supernatant that contains DNA and place into new microcentrifuge tubes.

**PCR reagents for a 10 μL reaction**

1. 5.0 μL of PCR MasterMix (Promega, Madison, Wisconsin, USA).
2. 2.5 μL of DNA-free H_2_O
3. 0.5 μL MgCl_._.
4. 0.5 μL forward primer.
5. 0.5 μL reverse primer.
6. 1.0 μL of template DNA.

**PCR cycling profiles**

CO1

94°C for 3 min, 10 cycles of 94°C for 50 s, an annealing temperature of 53°C that decreases by 0.5°C each cycle for 50 s, and 72°C for 1min 15s, followed by 30 cycles of 94°C for 50 s, 48°C for 50 s, and 72°C for 50 s, and a final extension period of 72°C for 5 min. Hold at 12 °C.

CAD, EF1-α, and H3

94°C for 3 min, 8 cycles of 94°C for 50 s, an annealing temperature of 57°C that decreases by 0.5°C each cycle for 50 s, and 72°C for 1min 15s, followed by 30 cycles of 94°C for 50 s, 53°C for 50 s, and 72°C for 50 s, and a final extension period of 72°C for 5 min. Hold at 12 °C.

DDC

94°C for 3 min, 8 cycles of 94°C for 50 s, an annealing temperature of 55°C that decreases by 0.5°C each cycle for 50 s, and 72°C for 1min 15s, followed by 30 cycles of 94°C for 50 s, 50°C for 50 s, and 72°C for 50 s, and a final extension period of 72°C for 5 min. Hold at 12 °C.

GAPDH

94°C for 3 min, 35 cycles of 94°C for 50 s, 52°C for 50 s, and 72°C for 1 min 30 s, and a final extension period of 72°C for 5 min. Hold at 12 °C.

DDC, GAPDH, and CAD interior primers

94°C for 3 min, 10 cycles of 94°C for 50 s, an annealing temperature of 60°C that decreases by 0.5°C each cycle for 50 s, and 72°C for 1min 15s, followed by 30 cycles of 94°C for 50 s, 55°C for 50 s, and 72°C for 50 s, and a final extension period of 72°C for 5 min. Hold at 12 °C.

**Modified EXO-SAP cleanup for 96 samples of 5-7 μL PCR product**

1. In a 0.5 mL tube combine the following:
   1. 156 μL ddH2O.
   2. 4.0 μL Exonuclease I (10 U/μL).
   3. 40 μL Shrimp Alkaline Phosphatase.
2. Gently vortex (200 μL total).
3. Add 2 μL EXO-SAP solution to 5 μL PCR product.
4. Incubate at 37 °C for 20 minutes.
5. Incubate at 80 °C for 15 minutes.

**Modified 10 μL BigDye® Terminator v3.1 (ThermoFisher) cycle sequencing reaction**

1. 4.0 μL DNA free H_2_O
2. 3.5 μL 2.5x BigDye® v3.1 reaction buffer
3. 0.5 μL BigDye® v3.1
4. 1.0 μL forward primer
5. 1.0 μL purified PCR product

**Thermocycler conditions for cycle sequencing.**

1. 96°C for 1 min
2. 25 cycles of:
   1. 96°C for 10 s
   2. 50°C for 5 s
   3. 60°C for 4 mins
3. Hold at 4°C.

**EtOH cleanup for 10 μL cycle sequencing product in a 96 well plate**

1. Add 2.5 μL 125mM EDTA to all wells containing sequencing product.
2. Add 30 μL 100% EtOH to all well containing sequencing product.
3. Seal plate and invert a few times to mix.
4. Leave at room temperature for 10 mins to precipitate extension products.
5. Pellet sequencing product in refrigerated centrifuge at 2500g for 30 minutes at 4 ºC.
6. Remove seal and invert well plate onto paper towel and secure with rubber bands.
7. Place well plate inverted into centrifuge and spin 60 g for 3 minutes. This will remove any EDTA and EtOH from the wells.
8. Repeat steps 2 to 7 to this time using 70% EtOH
9. Put tubes in 65 °C oven for 10 minutes to ensure EtOH evaporates completely
10. Cover in aluminum foil and store at 4.0 °C
