# Supplementary figures and images for: Phylogeny, host use, and diversification in the moth family Momphidae (Lepidoptera: Gelechioidea)
Source: PLoS One. 2019 Jun 6;14(6):e0207833. doi: 10.1371/journal.pone.0207833 (PMC6553701; doi:10.1371/journal.pone.0207833)

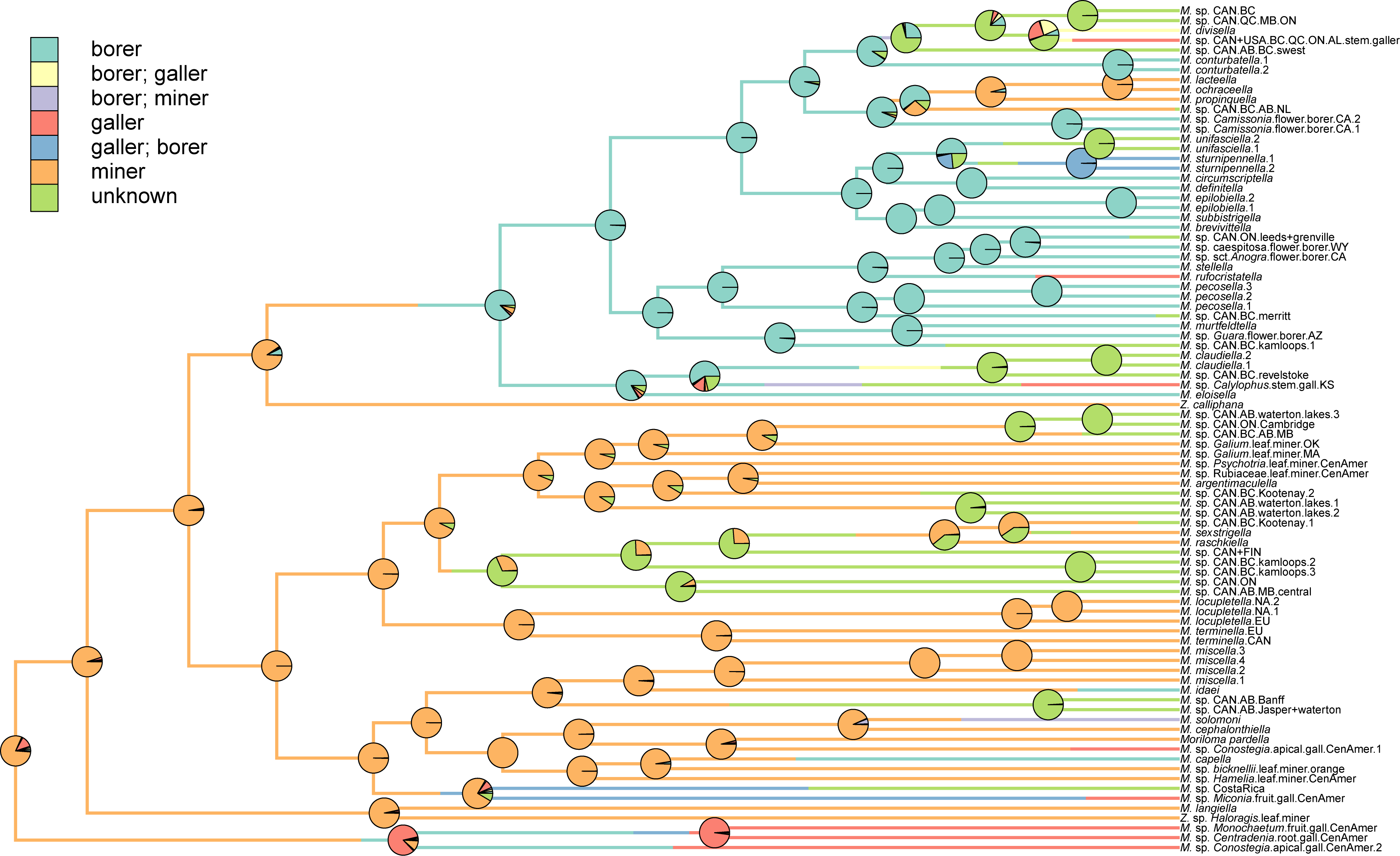

Supplement: S1 Fig — Stochastic character mapping with 10,000 replicates. Posterior probabilities at each node displayed as pie charts. Colors on branches and the pie charts represent most likely character state at each node. (TIF) [file pone.0207833.s011.tif]

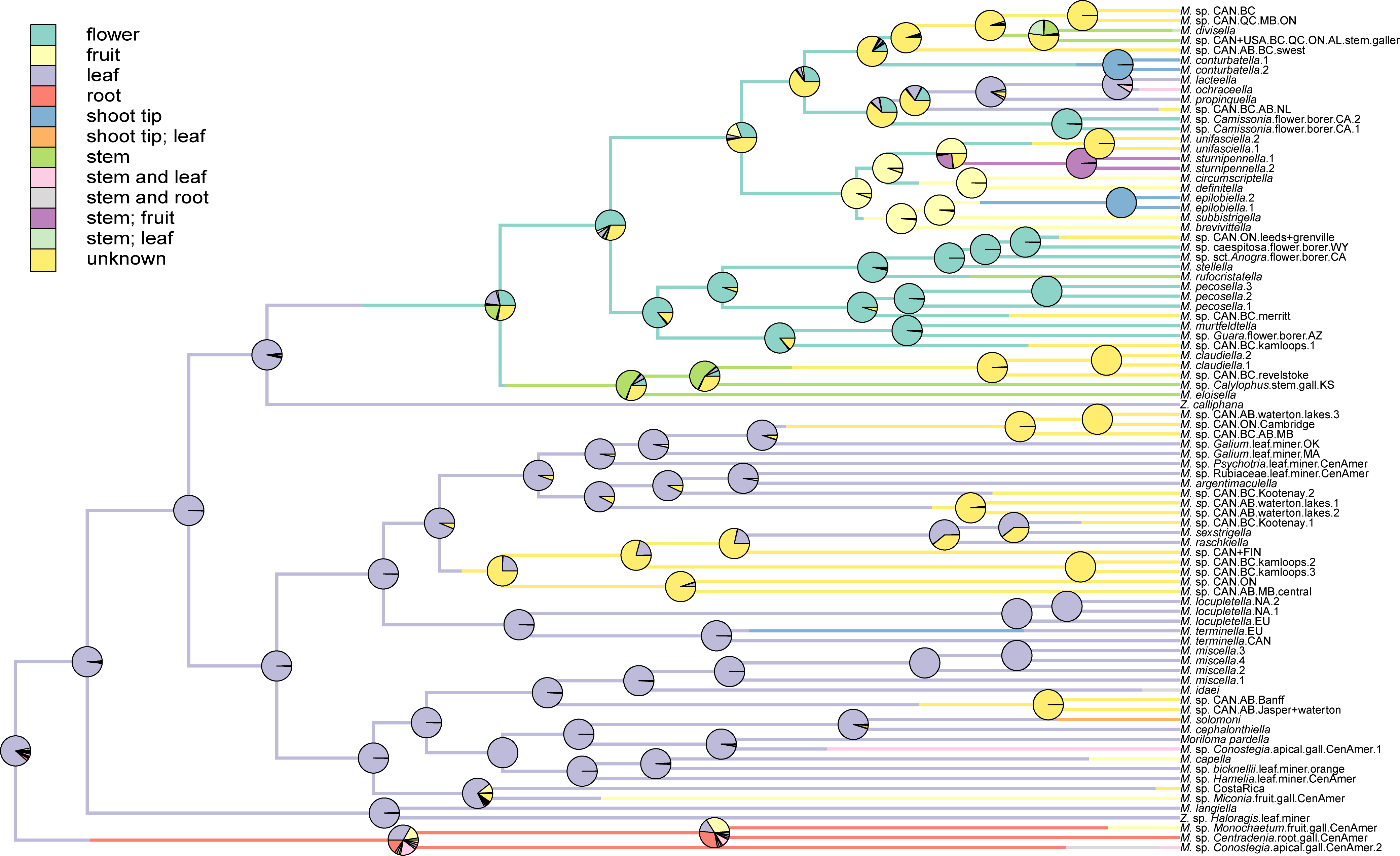

Supplement: S2 Fig — Stochastic character mapping with 10,000 replicates. Posterior probabilities at each node displayed as pie charts. Colors on branches and the pie charts represent most likely character state at each node. (TIF) [file pone.0207833.s012.tif]
